# Metformin Treatment in Hyperglycemic Critically Ill Patients: Another Challenge on the Control of Adverse Outcomes

**Published:** 2011

**Authors:** Yunes Panahi, Mojtaba Mojtahedzadeh, Nuria Zekeri, Fatemeh Beiraghdar, Mohammad-Reza Khajavi, Arezo Ahmadi

**Affiliations:** a *Chemical Injuries Research Center, Baqiyatallah Medical Sciences University, Tehran, Iran. *; b *Pharmaceutical Research Center, Tehran University of Medical Sciences, Tehran, Iran.*; c *Faculty of Pharmacy, Tehran University of Medical Sciences, Tehran, Iran.*; d *Nephrology and Urology Research Center, Baqiyatallah Medical Sciences University, Tehran, Iran.*; e *ICU, Sina Hospital, Tehran University of Medical Sciences, Tehran, Iran*; f *Trauma Research Center, Tehran University of Medical Sciences, Tehran, Iran.*

**Keywords:** Metformin, Hyperglycemic, Ill patients, Insulin.

## Abstract

New-onset hyperglycemia in patients admitted to intensive care units increases the risk of morbidity and mortality. Insulin resistance is frequently seen in the treatment of stress-induced hyperglycemia. Metformin, an oral anti-hyperglycemic agent, may introduce a new treatment protocol in critically ill patients with insulin-resistance hyperglycemia.

Fifty-one non-diabetic traumatized patients with blood sugar (BS) levels more than 130 mg/dLwere introducedto three days of treatment with intensive insulin (50 IU) or metformin (1000 mg, twice daily) therapy. Clinical evaluationsincluded acute physiological and chronic health evaluation (APACHE II) and Glasgow Coma Scale (GCS). Experimental tests included BS level, mean arterial pressure (MAP), pH, HCO_3_, and lactate.

Eight patients were excluded and 21 of remained patients treated with insulin and 23 with metformin. There was no significant difference in terms of the evaluated factors between the two groups at the time of admission. Although desirable BS level (BS < 130 mg/dL) was reached by three days of metformin treatment (p < 0.01),there was no significant difference in BS, MAP, pH and HCO_3_of insulin treated groupin comparison with metformin treated patients. The findings weresimilar for APACHE II and GCS as well.

Although obvious studies are required, these findings may lead to effective therapies against stress-induced hyperglycemia.

## Introduction

There is a high risk of death and substantial morbidity in critically ill patients who require intensive care (1), because of increased susceptibility to severe infections and failure of vital organs that amplify the risk of an adverse outcome (2). In these patients, as a normal response to stress, hyperglycemia is frequently seen. One third of all patients admitted to tertiary care centers have hyperglycemia, about 12% of whom have had no prior history of diabetes (3) that confirmsthe hyperglycemia of new onset in the general hospital wards(4) and intensive care units (5, 6).

Since thetraditional thought suggests hypoglycemia presents a more serious risk to critically ill patients than hyperglycemia really does,the treatment is often not initiated unless blood glucose exceeds 200 to 250 mg/dL(7, 8). Strict glucose control and safe implementation of normoglycemia in intensive care patients offers significant benefits for them (9, 10). Hyperglycemia in this population of patients derived from increased gluconeogenesis and insulin resistance (11, 12).

Insulin administration has been used in critically ill patients, other than hyperglycemic crises, to improve clinical outcomes (13). However, insulin resistance is a central feature of stress metabolism in postoperative patients, trauma patients, sepsis, and critical illness in general. In insulin resistance, glucose uptake is reduced in peripheral, insulin-sensitive tissues, whereas endogenous glucose production is increased, resulting in hyperglycemia (14).

Metformin was approved for patients with diabetes mellitus (15). Itsignificantly accelerates glucose clearance, thereby attenuating hyperglycemia (16, 17). During hyperinsulinemia, glucose uptake was significantly greater in metformin-treated patients (18, 19). Patients receiving metformin also had a significantly higher plasma concentration of insulin (20, 21).As the existence of insulin resistance is suggested even in non-obese patients, for example with a BMI of 25 (22, 23), metformin therapy can be considered for the treatment of such patients as well(24).In case of critically ill patients,insulin resistance is seen along with hyperglycemia;therefore, treatment with metformin may introduce new strategies in treatment of hyperglycemia and its adverse effects. The present studyevaluatesthe consequence of metformin treatment in hyperglycemic critically ill patients.

## Experimental

*Study population*

In this randomized clinical study, data was collected prospectively form non-diabetictraumatized patients admitted to the multi-disciplinary ICU of Sina Hospital(Tehran, Iran) with blood sugar levels more than 130 mg/dL. Between April 2006 and October 2007,fifty-one patients were eligible for enrollment in the study after their closest family members confirmed thewritten informed consent which they were given.Methods of data collection and analysis were approved by the research ethics committee of the hospital accredited by Ministry of Health and Medical Education of Iran.Eight patients were excluded in first 12 h of study due tothe severe hemodynamical instability or signs of exclusion criteria including:age less than 18 or more than 75 years old, chronic renal failure (CRF, Creatinine> 1.2 mg/dL), bicarbonate < 13 mEq/lit, lactate > 4.5 mmol/lit, mean arterial pressure (MAP < 70 mmHg), creatinine clearance < 70 mL/min, pH < 7.3, and diabetic patients.

*Study design*

At the time of admission to the intensive care unit, included patients were randomly assigned to receive one of two protocols A or B for 72 h. Then, patients received routine protocol.

*Protocol A: Intensive insulin administration through intravenous injection*

Fifty IU regular insulin (Exir Co., Tehran, Iran) wassolved in 50 mLsodium chloride 0.9% solution. The amount of insulin infusion started in accordance with first blood sugar (BS) and then each 2 h BSwas checkedusing glucometer (Roche ACCU-Check comfort or active system) to determine the requiredchanges on insulin infusion.

*Protocol B: Thousand mg of metformin (Exir Co., Tehran, Iran), twice daily through oral or NG tubeadministration*

Only in the case of TPN, these patients received insulin in accordance with the amount of calories that had received. In this protocol, if the amount of BS in two subsequent evaluations was more than 300 mg/dL, that patient excluded from study. Serum lactate was checked (Roche Accutrend lactate system) per 6 h and metformin administration was stopped if serum lactate level was more than 4.5 mmol/L or if it was 2mmol/L more than previous level, and also if HCO_3_< 13 mEq/L or pH<7.35 or serum creatinine remained above 1.2 mg/dL through two evaluations, and if MAP<70 mmHg or urinary volume was less than 0.5 mL/Kg/min. All the excluded patients retained to ordinary protocol.

*Measurement of APACHE II (acute physiological and chronic health evaluation) and GCS (glasgowcoma scale)*

APACHE II which is one of the scoring systems in ICU units, measures the severity of disease in scales between 0 and 71, where increasing of score shows severity of disease and risk of death. APACHE II derives from twelve routine physiological assessments including blood pressure, temperature, heart rate and *etc*.

GCS is an observational method toassessthe patient’s consciousness in the base of neurologic scale from grade 3 (deep coma or death) to 15 (complete consciousness). GSC includes three tests of eye, speech and motor responses(25).

*Statistical analysis*

SPSS software (Statistical Product and Services Solutions, version 13.0, SPSS Inc, Chicago, IL, USA) was used to analyze the data. Statistical differences between groups were assessed using the Mann-Whitney test, and the t-test for continuous data. Values ofp > 0.05 were considered statistically significant.

## Results and Discussion

Twenty-one patients were treated with insulin as protocol A and 23 of them were introduced to protocol B (metformin treatment). Mean age of insulin and metformin-treated patients was 50 ± 21.68 and 48.4 ± 16.77 respectively.

Initial experimental evaluations and clinical assessments weretaken from patients of two groups in the admission period (Table 1). 

**Table 1:** Data comparison between two groups of treatments before starting the insulin or metformin administration

	**Protocol A: Insulin treatment group**	**Protocol B: Metformin treatment group**	**p-value**
**Age **	50 ± 21.68	48.4 ± 16.77	
**BS (mg/dL)**	149.25 ± 10.31	177.81 ± 20.17	0.32
**MAP (mmHg)**	94.7 ± 12.15	86.9 ± 7.14	0.07
**HCO**_3 _ **(mEq/L)**	23.15 ± 2.30	23.80 ± 3.54	0.69
**pH**	7.42 ± 0.05	7.37 ± 0.11	0.15
**APACHE II**	23.25 ± 6.92	20 ± 6.32	0.42
**G.C.S**	9.5 ± 3.93	7.4 ± 3.78	0.20

Data are shown as mean ± SEM. All data have been taken before starting the treatment in both groups. P < 0.05 was assumed as statistical significance.

There was no significant difference between thefirst BS levelof theadmitted patients forthe two different protocols (p = 0.32). Moreover, mean arterial pressure (MAP), serum HCO_3 _and pH levels were not statistically significant betweenthe patients of two groups in admission (p= 0.07, 0.69 and 0.15 respectively, Table 1). In addition, APACHE II and G.C.S have not showed significant difference for admitted patients of both groups in first day before the treatment.

BS: Blood Sugar; MAP: Mean Arterial Pressure; APACHE II: Acute Physiological and Chronic Health Evaluation; G.C.S: Glasgow Coma Scale.

*Blood sugar monitoring for three days of treatment*

**Figure 1:**
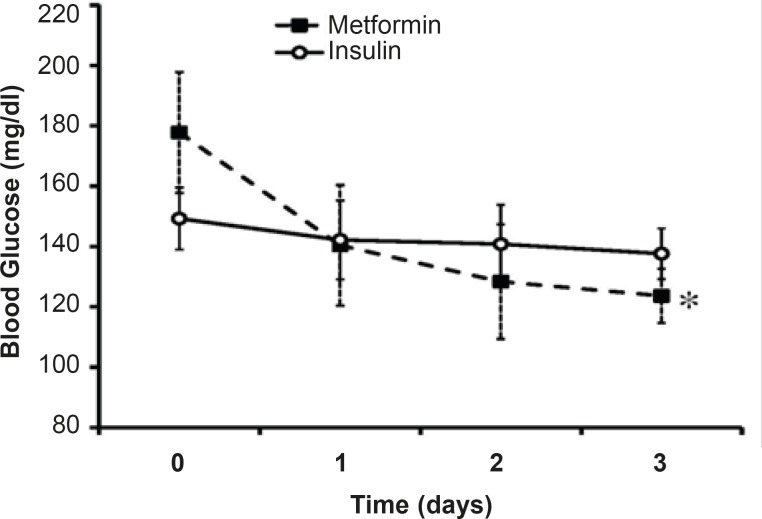
Effects of intensive insulin-therapy or metformin oral administration on blood sugar levels.Day 0 shows the BS levels before the beginning of each protocol. Data were expressed as mean ± SEM. *: p < 0.01 in comparison with BS levels obtained before the onset of metformin treatment

The mean levels of BS along three days of treatment were 140.19 ± 6.32 mg/dL in insulin-treated group and 130.77 ± 2.1 mg/dL in metformin-treated group. Although the mean level of BS in metformin-treated group was less thanthat of the other group, the difference was not significant (Figure 1). In insulin-treated group, the men’s level of BS in the 1^st^ day of admission was 149 mg/dL that reached to 137.61 mg/dLin the 3^rd ^day of insulin treatment (p=15). Metformin reduced BS from 177 mg/dL in the1^st ^day of admission to 123.63 mg/dL in the 3^rd ^day of treatment (p <0.01; Figure 1).

Desirable BS level (80 mg/dL<BS< 120 mg/dL) was not achievedthrough three days of insulin treatment (137.61 mg/dL) whileit reached near the normal range after three days of metformin treatment (123.63 mg/dL).

*Effects of different protocols on MAP*

**Figure 2:**
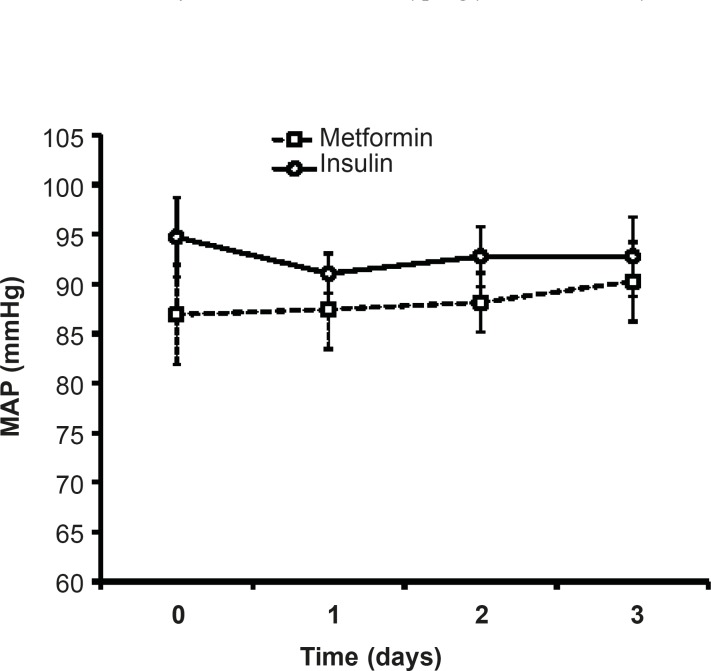
Mean arterial pressure (MAP) during three days of insulin or metformin treatment. Data were expressed as mean ± SEM

**Figure 3:**
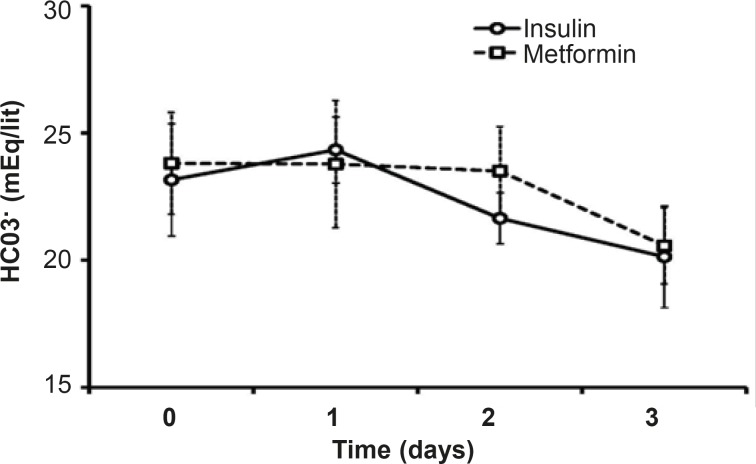
Changes on HCO3^-^serum during the treatment with insulin or metformin.Data were expressed as mean ± SEM

As shown in Figure 3, there was no significant difference in MAP of patients treated with insulin compared to metformin-treated patients.During three days of insulin or metformin treatment, MAP was not changed significantly in comparison with the admitted values (Figure 2).

*HCO*_3_ *and pH*

Both pH and HCO_3_ did not change statistically during theinsulin or metformin treatment. There is no significant difference between the mean levels of HCO_3_in the mentioned groups duringthe treatment days or in each day of treatment (Figure 3).The mean pH level of metformin treated patients (7.38 ± 0.32) was less than that of insulin group (7.43 ± 0.29), which was not significant.

*Serum’s lactate in metformin-treated patients*

**Figure 4:**
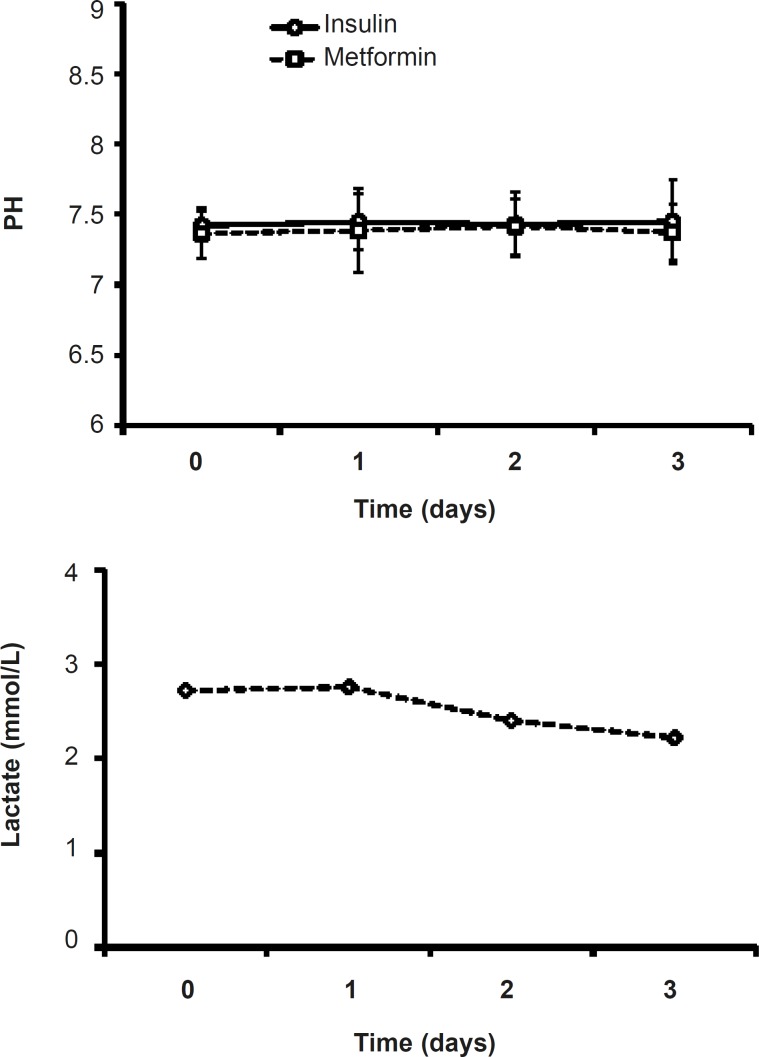
Effects of insulin or metformin treatment on the pH of serum. Data were expressed as mean ± SEM

**Figure 5:**
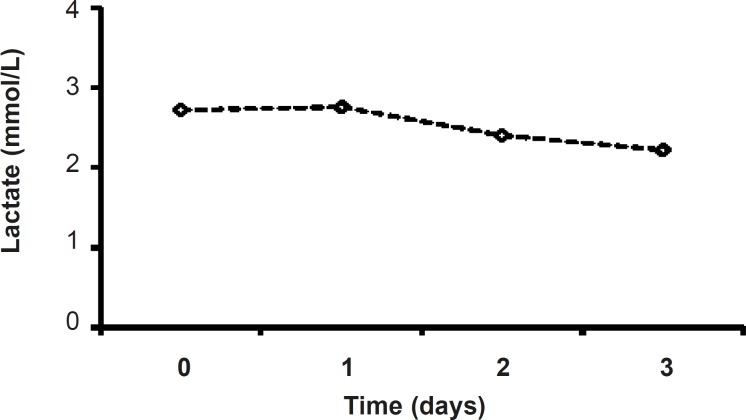
Serum lactate levels during three days of metformin treatment (1000 mg, twice daily).

Although in metformin-treated patients, the level of lactate at the end of procedure (2.22 ± 0.31) was less than that of the admitted point lactate value (2.87 ± 0.38), there were no significant changes in lactate values of metformin-related group during the study (Figure 5). (Figure 4).

*Clinical assessments including APACHE II and G.C.S scale*

**Figure 6:**
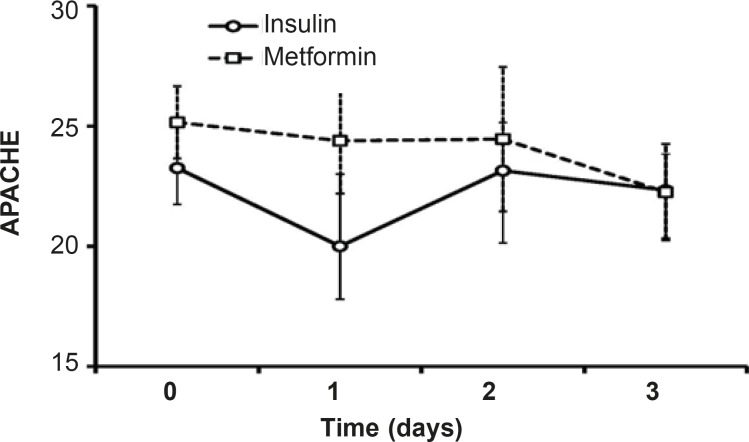
Evaluation of APACHE II (acute physiological and chronic health evaluation) as an index of severity of disease in scales between 0 and 71.Data were shown as mean±SEM during three days of insulin or metformin treatment and before the beginning of each treatment (Day 0).

Along three days of treatment, APACHE II did not change significantly in insulin-treated group or in patients treated with metformin. Although the level of APACHE II in protocol B patients was high than insulin-treated patients, the difference was not significant and afterthree days of treatment, the mean value of APACHE II was the same in both groups (22.^25^, Figure 6).

**Figure 7:**
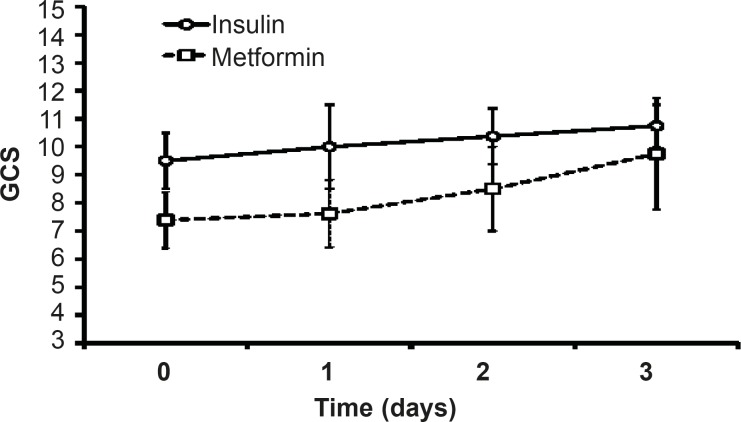
Glasgow Coma Scale (G.C.S) used for assessing the patients’ consciousness in scales between 3 (deep coma or death) to 15 (complete consciousness). Data were shown as mean±SEM before the beginning of each treatment (Day 0) and during three days of insulin or metformin treatment

G.C.S value increased after three days of insulin or metformin treatment but there was no significant difference between themin both groups. The value of G.C.S during three days of insulin-treatment was more than the values on metformin-treated patients, but statistical analysis did not showed any significant difference between the groups (Figure 7).

Hyperglycemia adversely affects the fluid balance, predisposes patient to infection, and increases the risk of renal failure, polyneuropathy and mortality in ICU (26). Although it has been reported that low dose metformin therapy improvesthe glycemic control in type 2 diabetic patients (24, 27),there has been no study so far comparing the effects of metformin therapy with that of insulin on hyperglycemia treatmentin critically ill patients. The currentstudywas aimed tocompare the effects of metformin and intensive insulin administration on the hyperglycemia treatment in critically ill patients for the first time and showed that the metformin treatment reduces BS levels near to normal range. However, there was no difference in MAP, HCO_3 _and pH of insulin and metformin-treated patients. Both insulin and metformin treatmentsleaded to non-significant increase in GCS values and decrease in APACHE II.

A recent study on the comparison of metabolic effects of insulin and metformin on patients with severe burn injury greater than 40% of their body surfaceshowed a significant anabolic effect on muscle protein with metformin and a modest response with insulin and suggested that metformin and insulin may work synergistically to further improve on muscle protein kinetics (28).In present study, metformin therapy reduced the BS levels but could not induce significant change on other evaluated parametersduring the treatment period and results werethe same as insulin.

It has been demonstrated that metformin reduces blood glucose levels predominantly through improving hepatic and peripheral tissue sensitivity to insulin without affecting the secretion of this hormone.It has been shown that metformin does not cause hypoglycemia(29).Metformin plus insulin appear to lower the incidence of insulin resistance and insulin requirement while maintaining blood glucose level control and consequently lower the incidence of adverse effects related to high-dose insulin therapy, particularly hypoglycemia in patients admitted to ICU(30, 31). In a study by Ansari G.*et al.*, patients with systemic inflammatory response syndrome (SIRS) and a blood glucose level of more than 120 mg/dLwere admitted to an ICU received intensive insulin therapy or combination therapy with metformin andthen blood samples were obtained at baseline and at 48 h, 96 h and 7 days after the initiation of study. The results of this study revealed that the addition of metformin to the insulin decreased insulin requirement and concentration of insulin and C-peptide, whereas decreased blood glucose level, therefore lowered the incidence of adverse effects related to the high-dose insulin therapy (31). A review of study (based on EMBASE and MEDLINE searches from January 1990 to April 2006) done by Staels on two key classes of insulin-sensitizing agents–the biguanides (principally metformin) and thiazolidinediones (pioglitazone and rosiglitazone)–suggested that the thiazolidinediones and metformin, in spite of their distinct mechanisms of action, can provide the clinical opportunity for effective glucose control and metabolic risk reduction (31).

We did not used combination therapy of insulin and metformin sincethe aim ofpresent study was the comparison of two different treatments separatelyand the resultshowedthat when metformin is used as a single treatment,it canreduce the BS levels. To understand the preciseeffect of single metformin treatment on hyperglycemic patients in ICU, further studies on large ranges of samples and designing advanced protocols is needed.

Lactic acidosis caused by metformin is rare and the risk of this complication may be diminished by the observance of prescribing precautions and contraindications that avoid accumulation of metformin or lactate in the body (15).Lactateand pH levels did not change significantly during three days of treatment (Figures 4 and 5) which is a proofthe fact thatmetformin (1000 mg/day)cannot induce lactic acidosis.

In conclusion, compared to the intensive insulin therapy, metformin could not induce a significant change on the experimental parameters or conscious statesof critically ill patients. Although metformin reduced BS levels to desired values along three days of treatment,further studies are neededtoencompass more patients orcombination therapies with insulin or evaluation for a long time todisclose possible clinical insights on reducing the critically ill patients’ mortality.
